# Gut microbiota dysbiosis exacerbates heart failure by the LPS-TLR4/NF-κB signalling axis: mechanistic insights and therapeutic potential of TLR4 inhibition

**DOI:** 10.1186/s12967-025-06821-8

**Published:** 2025-07-10

**Authors:** Chunlei Zhang, Xiaodong Teng, Qiuhang Cao, Yanyan Deng, Mo Yang, Lei Wang, Daorong Rui, Xiu Ling, Cao Wei, Yue Chen, Dasheng Lu, Hongxiang Zhang

**Affiliations:** 1 The Second Affiliated Hospital of Wannan Medical College Department of Cardiology, Wuhu, AnHui China; 2 Department of Respiratory Disease, Shangqiu Municipal Hospital, Shangqiu, Henan China; 3 Vascular Diseases Research Center of Wannan Medical College, Wuhu, AnHui China; 4 Wuhu Vascular Disease Technology Research and Development Center, Wuhu, AnHui China

**Keywords:** Gut microbiota, Heart failure, Lipopolysaccharide, TLR4/NF-κB signalling pathway, Phosphorylated p65 protein

## Abstract

**Objective:**

This study aimed to investigate the associations between gut microbiota dysbiosis and alterations in cardiac function and to elucidate the underlying molecular mechanisms involved.

**Methods:**

Eighteen rats were divided into a control group (*n* = 6), a heart failure (HF) group (*n* = 6), and a TAK-242 intervention group (*n* = 6). Cardiac function was assessed using small-animal echocardiography. Serum levels of brain natriuretic peptide (BNP) and inflammatory cytokines were measured by ELISA. Western blotting was used to detect phosphorylated p65 (P-p65) protein expression in myocardial tissue, and 16 S rRNA sequencing was performed to analyse the composition of the faecal gut microbiota.

**Results:**

Compared with the control group, the heart failure group presented significant gut microbiota dysbiosis, characterized by increased relative abundance of Bacteroidetes and Spirochaetes and decreased relative abundance of Actinobacteria and Proteobacteria, along with reduced species diversity. The serum levels of lipopolysaccharide (LPS), IL-1β, IL-17, IL-6, and TNF-α were significantly elevated (*P* < 0.05). Myocardial tissue pathology revealed disordered myocardial fibre arrangement and significant lymphocyte infiltration. TAK-242 intervention normalized the gut microbiota composition; reduced LPS and inflammatory cytokine levels; improved the left ventricular ejection fraction (LVEF) and left ventricular fractional shortening (LVFS); and decreased the left ventricular end-systolic diameter (LVESD), left ventricular end-diastolic diameter (LVEDD), and BNP levels. Myocardial tissue pathology also improved. Western blot analysis revealed increased TLR4 、P-IKBα/ IKBα and P-p65/p65 expressions in the heart failure group, which were significantly inhibited by TAK-242 (*P* < 0.05).

**Conclusion:**

Gut microbiota dysbiosis exacerbates heart failure by activating myocardial inflammation through the LPS-TLR4/NF-κB signalling pathway. By modulating this pathway, TAK-242 improves cardiac function, suggesting its potential as a therapeutic target for heart failure.

**Clinical trial number:**

Not applicable.

**Supplementary Information:**

The online version contains supplementary material available at 10.1186/s12967-025-06821-8.

## Introduction

Despite significant advancements in modern medical therapies for heart failure (HF), including standardized pharmacological treatments and improved interventional procedures, the mortality rate among HF patients remains alarmingly high. This underscores the limitations in our understanding of the underlying mechanisms of this disease. In recent years, accumulating evidence has highlighted the pivotal role of chronic immune system activation and sustained dysregulation of inflammatory responses in the pathological progression of HF [1]. As a critical defence system for maintaining homeostasis, the immune system plays dual roles in cardiac development, tissue repair, and remodelling: moderate immune responses facilitate tissue repair and functional preservation, whereas chronic immune activation leads to maladaptive inflammation and tissue fibrosis [2], ultimately contributing to impaired cardiac systolic and diastolic function [3].

Within this complex pathological framework, Toll-like receptor 4 (TLR4), a key pattern recognition receptor in the innate immune system, has emerged as a central player in the pathogenesis of cardiovascular diseases [4–6]. TLR4 specifically recognizes pathogen-associated molecular patterns and activates the nuclear factor-κB (NF-κB) signalling pathway, triggering the cascading release of inflammatory cytokines and amplifying persistent inflammatory responses [7]. Previous meta-analyses have confirmed the critical involvement of the NF-κB signalling pathway in HF [8, 9], providing crucial insights into the immunological mechanisms underlying this condition.

Notably, recent studies have focused increasing attention on the relationship between the gut microbiota and cardiovascular diseases [10–12]. As the largest microbial ecosystem in the human body, the gut microbiota participates in diverse physiological processes [13] and is closely linked to the development and progression of chronic heart failure (CHF) [14]. Research indicates that HF patients commonly exhibit intestinal barrier dysfunction, with elevated circulating levels of proinflammatory cytokines correlating significantly with symptom severity and a poor prognosis [15]. In HF, venous congestion, sympathetic activation, and reduced cardiac output collectively contribute to intestinal wall oedema and mucosal hypoperfusion [16], leading to impaired intestinal barrier integrity. This allows lipopolysaccharide (LPS), a component derived from gram-negative bacteria, to enter the systemic circulation. By binding to TLR4 on immune cells, LPS triggers the release of proinflammatory cytokines, culminating in systemic inflammation [17]. Specifically, LPS produced by gram-negative bacteria such as Enterobacteriaceae and Bacteroidetes promotes the secretion of proinflammatory mediators (e.g., IL-1, IL-6, and TNF-α) by the TLR4/NF-κB pathway in macrophages and dendritic cells, exacerbating endotoxaemia [10]. Previous studies have focused on the treatment of HF through dietary interventions [18], prebiotic and probiotic supplementation [19, 20] and gut microbiota transplantation [21] to intervene in disturbances of the intestinal flora. However, few interventions have targeted the molecular mechanisms underlying the relationship between disturbances in the gut flora and the development of HF.

On the basis of the above evidence, we hypothesized that gut microbiota dysbiosis promotes HF pathogenesis by the release of LPS, which activates the TLR4/NF-κB signalling pathway to induce inflammatory cytokine release, myocardial inflammation, and fibrosis. Blocking the interaction between TLR4 and LPS or inhibiting TLR4/NF-κB signalling may serve as a potential therapeutic strategy for modulating gut microbiota dysbiosis and treating HF. This study aimed to validate this hypothesis, offering a theoretical foundation and novel therapeutic method for HF.

## Materials and methods

### Experimental animals

Thirty SPF-grade male Sprague‒Dawley rats (weight: 200 ± 20 g) were purchased from the Qinglongshan Animal Breeding Center (Jiangning District, Nanjing, Jiangsu Province; animal production licence no. SCXK(Zhe)2019-0002). All the rats were housed under standard conditions (temperature: 22 ± 2 °C; humidity: 50 ± 10%; 12-hour light/dark cycle) with free access to food and water. A seven-day acclimatization period was provided prior to the experiments. The experimental protocol was approved by the Animal Ethics Committee of the Second Affiliated Hospital of Wannan Medical College (approval no. WYEFYLS 202012). All procedures conformed to the guidelines from Directive 2010/63/EU of the European Parliament on the protection of animals used for scientific purposes.

### Drugs and reagents

The drugs and reagents used were as follows: isoprenaline hydrochloride (ISO, MedChemExpress, cat. no. HY-B0468); TAK-242 (TLR4 inhibitor, MedChemExpress, cat. no. HY-11109); ELISA kits for serum brain natriuretic peptide (BNP), LPS, IL-1β, IL-6, IL-17, and TNF-α (Shanghai Weiao Biotechnology Co., Ltd., cat. nos. VA-EL-R0012, VA-EL-R0045, etc.); An HE staining kit and Masson’s trichrome staining kit (Servicebio, Wuhan, China; cat. nos. G1005, G1345).

### Instruments

The instruments used were as follows: a small-animal ultrasound imaging system (Vevo 3100, VisualSonics, Canada); an optical microscope (Nikon Eclipse E100, Japan); electrophoresis and transfer systems (Bio-Rad, USA); and a chemiluminescence imaging system (Tanon 5200, China).

### Establishment of a heart failure model using ISO and intervention with TAK-242

A total of 30 rats were selected, of which 24 were used to model heart failure and 6 were used as controls. The experimental rats were acclimatized to the environment in which they were housed for 7 days, and CHF model rats were generated by the intraperitoneal injection of ISO. Prior to the start of the study, we referred to the literature [22, 23] on the ISO-induced heart failure model in rats and chose a high dose (10 mg/kg/d) of ISO [24] administered intraperitoneally; however, the rats appeared to be curled up in the corner of the cage, walked unsteadily, and often died minutes after ISO was injected. Thus, we chose to use a low dose of ISO (3 mg/kg/d), which was administered for 14 days. During the administration of ISO, the rats were closely observed for changes in mental status, exercise, food intake and fur glossiness. The mortality rate of the rats was 50% (12/24) after the first injection of ISO, mostly within 15 min. Surviving rats gradually exhibited depression, decreased activity, decreased feeding, and yellowing of fur, and some rats developed diarrhoea. Moreover, 6 rats in the control group were intraperitoneally injected with equal volumes of normal saline for 14 days. After the last injection was completed, the following cardiac function indices were measured using small-animal ultrasound: left ventricular ejection fraction (LVEF), left ventricular short-axis shortening rate (LVFS), left ventricular end-systolic diameter (LVESD), and left ventricular end-diastolic diameter (LVEDD), followed by tail vein blood sampling to measure the serum BNP concentration. Compared with that in the control group, the LVEF was significantly lower, the serum BNP level was significantly greater, indicating successful modelling.

**Fig. 1:**
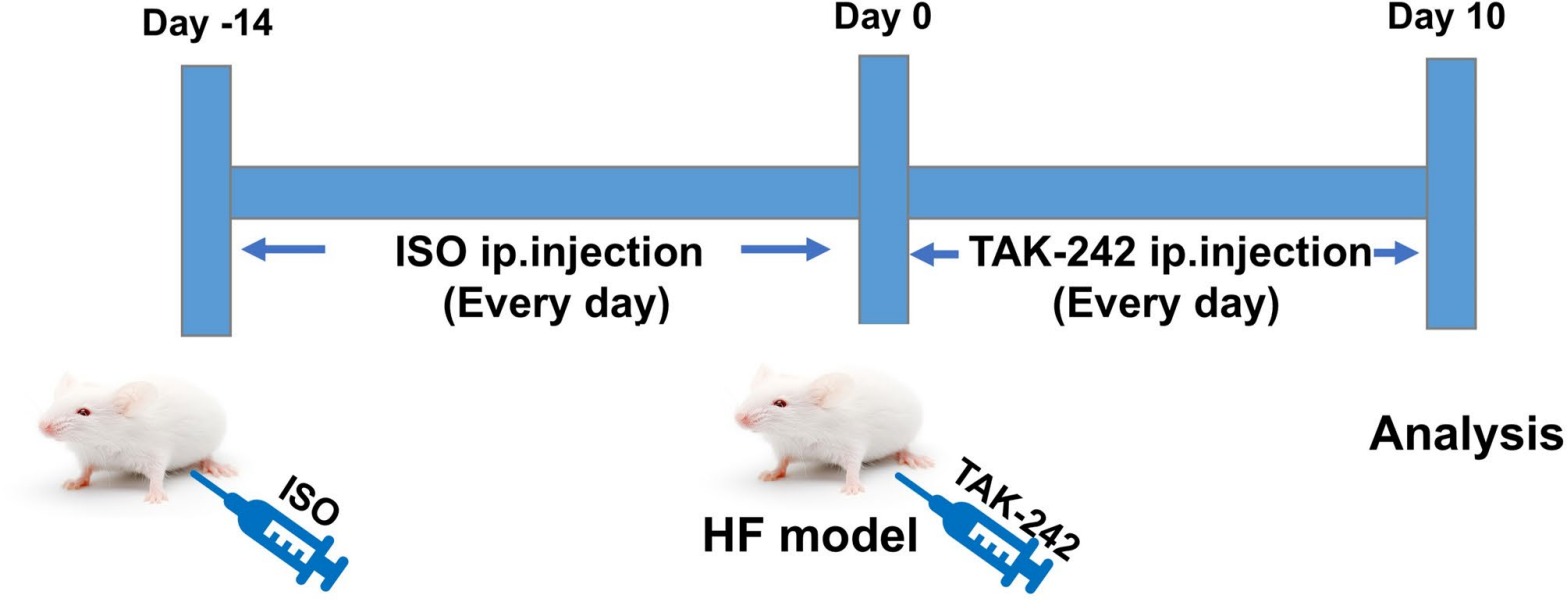
The schedule of rat heart failure modeling (ISO 3 mg/kg/d) and TAK-242 (2 mg/kg/d) intervention in rat heart failure

The 12 surviving rats with heart failure were randomly divided into a heart failure group (*n* = 6) and a TAK-242 group (*n* = 6). The TAK-242 group was given intraperitoneal injections of TAK-242 (2 mg/kg/day) [24, 25] for 10 days. Rats in the heart failure group (*n* = 6) and the control group (*n* = 6) were injected with an equal volume of saline for 10 days. Compared with the predose period, after 10 days of injections, rats in the TAK-242 group showed significant improvement in mental status, activity, and feeding; the fur became shiny, and the diarrhoea ceased. On the other hand, rats in the heart failure group exhibited further deterioration in their mental state, reduced food intake, and worse diarrhoea and huddled in the corner of the feeding cage, rarely moving. No deaths occurred in the three groups of rats during this period. We re-measured the following cardiac function indicators using ultrasound in three groups of rats: LVEF, LVFS, LVESD, LVEDD, and serum BNP levels. Figure 1 showed the schedule of the rat heart failure model (ISO 3 mg/kg/d) and TAK-242 (2 mg/kg/d) intervention.

### Sample collection and processing

#### Blood samples

After the HF model was established, 1 ml of blood was drawn from the tail vein of each rat in the HF group and the normal control group, after which it was centrifuged (3000 rpm, 15 min), and the serum was stored at -80 °C. Similarly, after 10 days of TAK-242 drug treatment in rats with heart failure, 1 ml of blood was withdrawn from the tail vein of each rat in the heart failure group, the TAK-242 group and the normal control group, after which it was centrifuged (3000 rpm, 15 min), and the serum was stored at -80 °C.

#### Cardiac tissue

At the end of the experiment, each rat was sacrificed by bloodletting under isoflurane anaesthesia, and the heart was quickly removed, washed with normal saline, and divided into two parts: A portion of myocardial tissue was placed in 4% paraformaldehyde fixation for 24 h and then preserved by embedding in a wax block to produce later sections for pathological staining, the other portion was rapidly frozen using liquid nitrogen and then preserved for subsequent western blot analysis.

#### Faecal samples

After 10 days of TAK-242 treatment in rats with heart failure, stool samples were collected from rats in the TAK-242 group, heart failure group and normal control group. Stool samples were snap-frozen and stored at -80 °C for 16 S rRNA sequencing.

### Detection methods

#### Echocardiography

After the rats were anaesthetized by inhalation of a mixture of 4% isoflurane and air, continuous inhalation anaesthesia was maintained with a mixture of 2% isoflurane and air. The anaesthetized rats were fixed in the supine position on the operating table, the chest hair of each rat was shaved, and a coupling agent was evenly applied to the chest. The probe of the diagnostic colour Doppler ultrasound instrument was placed on the left chest, and the long axis of the left ventricle was shown using two-dimensional echocardiography, with the apex of the heart at the same level as the outflow tract. The probe was rotated clockwise 90° to locate the maximal section of the left ventricle, and images were acquired. Using image analysis software, the epicardial and endocardial trajectories of the anterior wall of the left ventricle were traced, the posterior wall of the left ventricle was traced in the same way, and three consecutive cardiac cycles were traced to obtain the LVEF, LVFS, LVESD, and LVEDD.

#### ELISA

After ultrasound examination, the tail vein of each rat was disinfected, 1 ml of blood was drawn with a 1-ml syringe, the serum was centrifuged and separated, and the serum BNP and inflammatory factor levels were determined in strict accordance with the instructions of the ELISA kits.

#### Histopathology

At the end of the experiment, after anaesthesia with a mixture of 4% isoflurane and air, each rat was sacrificed by bloodletting. The heart was rapidly removed and subjected to HE staining for examination of myocardial structure and inflammation and Masson’s trichrome staining for examination of collagen (ImageJ analysis).

#### Western blotting

Ventricular tissue samples were homogenized in lysis buffer (Beyotime Biotechnology, Shanghai, China). Following centrifugation, protein concentrations in the supernatant were quantified using established protocols [26]. For Western blot analysis, the following primary antibodies were employed: Phospho-NF-κB p65 (ab76302, Abcam; 1:1000), Phospho-IκBα (ab133462, Abcam; 1:1000), TLR4 (ab22048, Abcam; 1:1000), IκBα (ab133462, Abcam; 1:1000), Total NF-κB p65 (ab76302, Abcam; 1:1000). β-actin (WA-1001, Weiao Biotechnology, Shanghai, China; 1:1000) served as the loading control for cytoplasmic proteins, while Lamin B1 (Santa sc-365962,1:700) was used for nuclear protein normalization.

#### Gut microbiota sequencing

16 S rRNA gene sequencing was conducted by Hangzhou Cosmos Wisdom. Fresh faecal tissues (from six rats in each group) were collected, and total DNA was extracted from the samples. Primers were designed according to the conserved regions, and a sequencing connector was added at the end of the primers. PCR amplification was carried out, and the products were purified, quantified, and homogenized to form sequencing libraries, which were then sequenced using an Illumina NovaSeq 6000 after quality control. The species composition of the samples was revealed by splicing and filtering the reads, clustering or denoising, and performing species annotation and abundance analysis. Beta diversity, significant species difference, correlation, and functional prediction analyses were further performed to examine the differences between samples.

### Statistical analysis

The numerical data for each group are expressed as the mean ± standard deviation (x̄±s). Statistical analysis of the experimental data was performed using SPSS 26.0 software. Significance testing between two groups was conducted using the t test, and comparisons between multiple groups were performed using one-way analysis of variance. *P* < 0.05 was considered statistically significant.

## Results

### Evaluation of the isoproterenol hydrochloride-Induced HF rat model

**Fig. 2:**
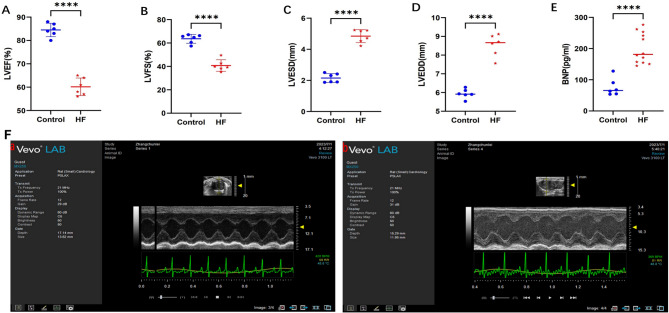
Cardiac function indices in ISO-induced heart failure rats. (**A**) LVEF was reduced in HF group; (**B**) LVFS was decreased in HF group; (**C**) LVESD was increased in HF group; (**D**) LVEDD was increased in HF group; (**E**) BNP was elevated in HF group; (**F**) Representative cardiac ultrasound images: a, control group; b, heart failure group; LVEF: left ventricular ejection fraction; LVFS: left ventricular fractional shortening; LVESD: left ventricular end-systolic diameter; LVEDD: left ventricular end-diastolic diameter; BNP: brain natriuretic peptide. *n* = 6 in each group, ****P < 0.0001

During the preparation of the rat HF model, deaths occurred primarily within 15 min after isoproterenol (ISO) administration, likely due to malignant arrhythmias caused by the ISO concentration and injection speed. The survival rate of the rats during model preparation was approximately 50%, whereas no deaths occurred in the control group. The day after the end of the 14-day period of ISO dosing, 6 out of the 12 surviving rats were randomly selected for echocardiography, and plasma BNP levels were measured in all surviving rats. Compared with control rats, ISO-induced HF rats presented significantly decreased LVEF and LVFS and increased LVESD, LVEDD, and BNP levels (Fig. 2). The differences were statistically significant (*P* < 0.05), indicating successful establishment of the HF model.

The low survival rate (50%) during model preparation may be attributed to the ISO concentration. Future experiments should reduce the ISO concentration to improve survival rates.

### Relationship between cardiac function changes and gut microbiota alterations in HF rats

**Fig. 3:**
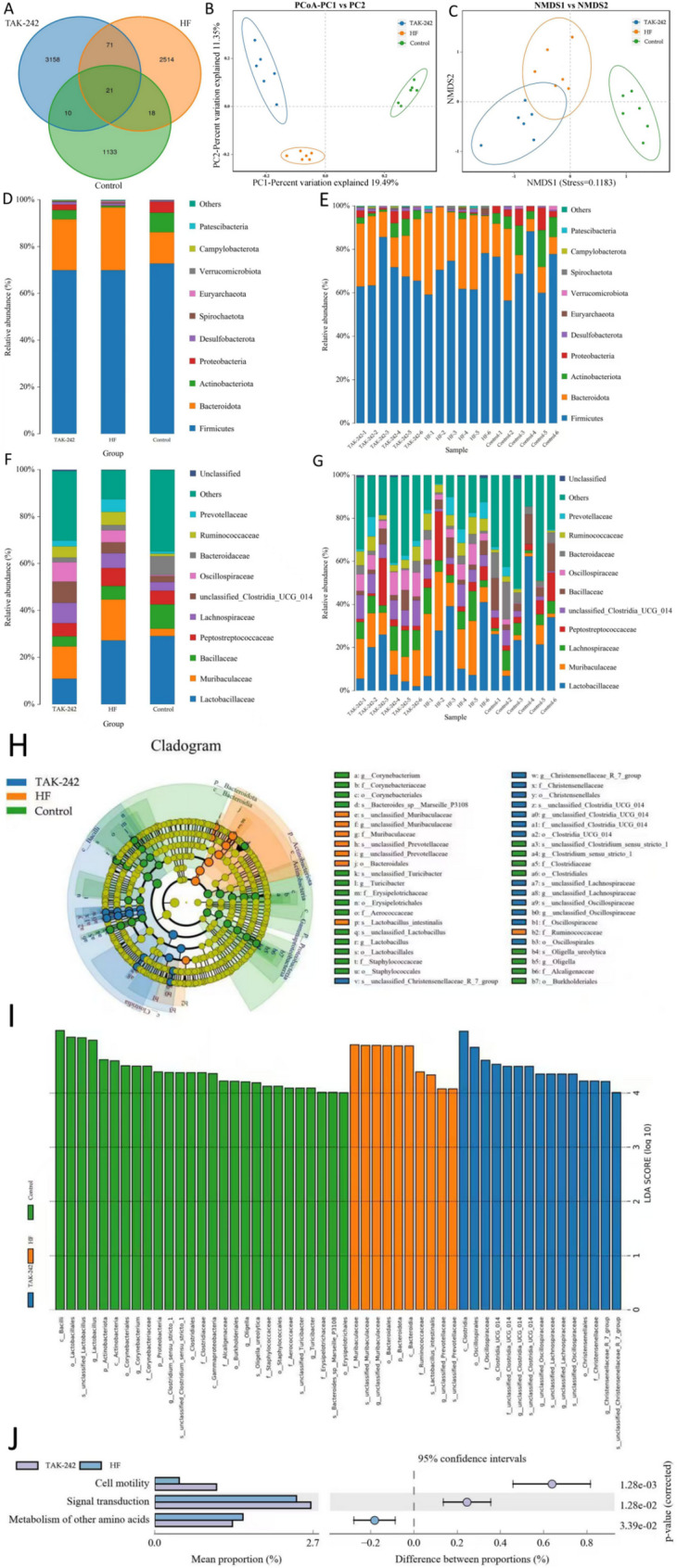
Effects of TAK-242 on gut microbiota dysbiosis in heart failure rats. (**A**) Venn diagram of OTUs and average overlapping OTUs among groups; (**B**) PCoA analysis; (**C**) NMDS analysis; (**D**) Phylum-level microbial composition in the three groups; (**E**) Phylum-level community bar plot in each of the animals; (**F**) Family-level microbial composition in the three groups; (**G**) Family-level community bar plot in each animal; (**H**, **I**) LEfSe analysis revealing the main differential microbiota among the three groups. Blue represents the microbiota that is relatively enriched in the TAK-242 grou; (**J**) KEGG pathway differences between TAK-242 and heart failure group

The gut microbiota of the rats was analysed using 16 S rRNA sequencing. Venn diagrams were used to visualize the shared operational taxonomic units (OTUs) and average OTU numbers among the groups (the TAK-242 group, HF group, and control group) (Fig. 3A). Principal coordinate analysis (PCoA) and nonmetric multidimensional scaling (NMDS) revealed significant differences in the gut microbial composition between the HF and control groups. After 10 days of TAK-242 treatment, the gut microbiota profile of HF rats showed marked improvement (Fig. 3B, C). At the phylum level, HF rats presented increased abundance of *Bacteroidota* and *Spirochaetota* but decreased abundance of *Actinobacteriota* and *Proteobacteria*. TAK-242 treatment reversed these changes (Fig. 3D, E). At the family level, *Muribaculaceae*, *Peptostreptococcaceae*, *Prevotellaceae* and *Ruminococcaccac* were enriched in the HF group, accompanied by reduced microbial diversity. TAK-242 treatment also reversed these alterations (Fig. 3F, G). Linear discriminant analysis (LDA) effect size (LEfSe) analysis revealed increased abundance of *Muribaculaceae*, *Bacteroidota*, and *Ruminococcaceae* in the HF group (Fig. 3I), and these increases were significantly reversed by TAK-242. Additionally, TAK-242 increased the abundance of *Clostridia* and *Oscillospiraceae* (Fig. 3H-I). KEGG pathway analysis revealed significant differences between the TAK-242 and HF groups in terms of “cell motility,” “signal transduction,” and “metabolism of other amino acids” (Fig. 3J).

These alterations in the gut microbiota may exacerbate myocardial injury by promoting the release of inflammatory factors. Prior studies have suggested a close link between gut dysbiosis, inflammation, and myocardial damage [12, 13]. Thus, TAK-242 likely improves cardiac function by restoring the gut microbiota balance and attenuating inflammation.

### Alterations in cardiac function and structure in rats

**Fig. 4:**
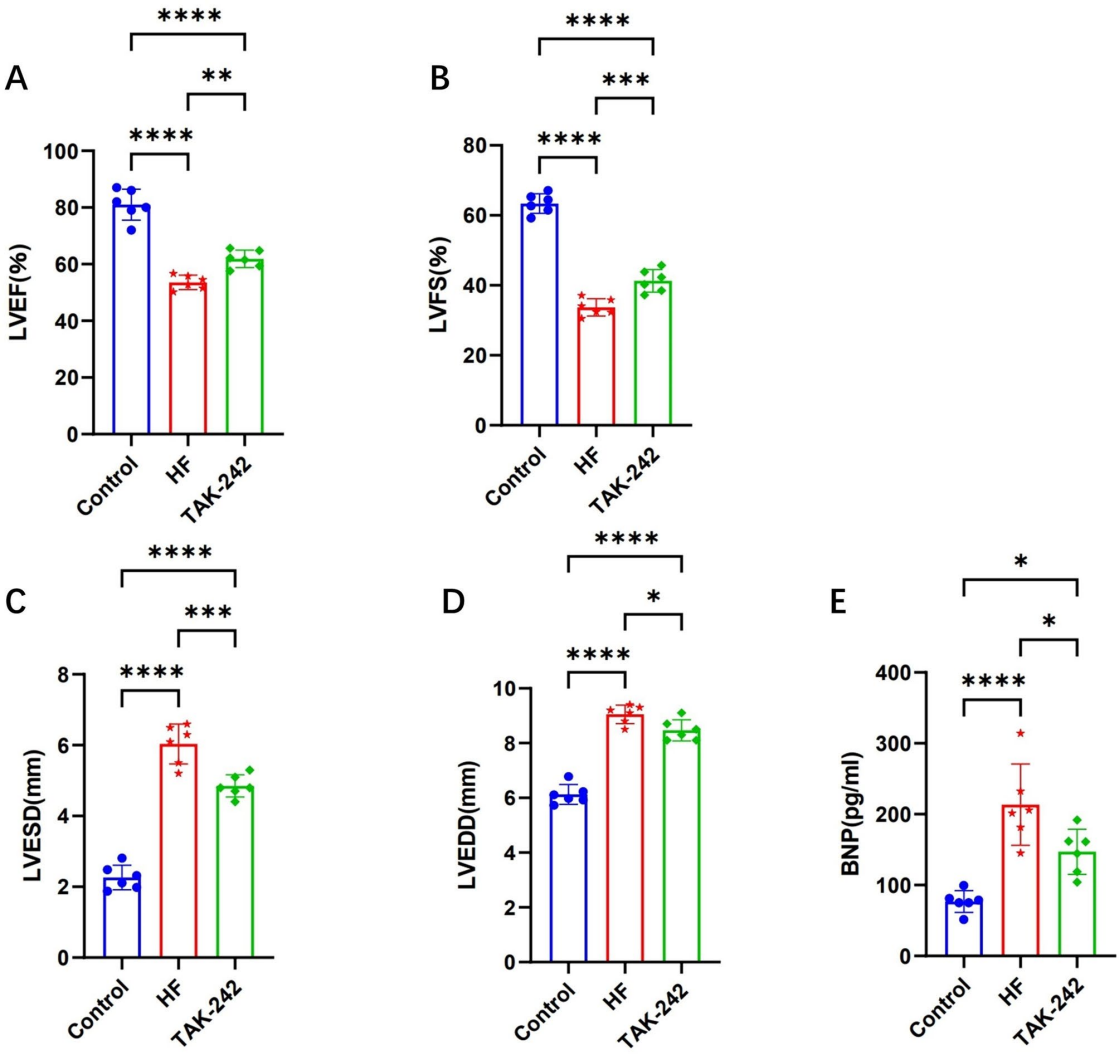
Cardiac function indices in TAK-242-treated rats. (**A**) LVEF and (**B**) LVFS were reduced in HF group, while TAK-242 treatment increased LVEF and LVFS. (**C**) LVESD and (**D**) LVEDD were increased in HF group, and reduced following TAK-242 treatment. (**E**) Serum BNP level was elevated in HF group, TAK-242 treatment decreased BNP level. *N* = 6 in each group, *P < 0.05, **P < 0.01, ***P < 0.001, ****P < 0.0001

After 10 days of TAK-242 treatment, cardiac structure and function were improved in rats. Compared with the HF group, the TAK-242-treated group presented increased LVEF and LVFS (Fig. 4A, B) and decreased LVESD, LVEDD, and BNP levels (Fig. 4C, D, E), indicating improved cardiac function. These results suggest that improved cardiac function is correlated with gut microbiota restoration.

### Changes in serum inflammatory factors, LPS levels, and myocardial histopathology

**Fig. 5:**
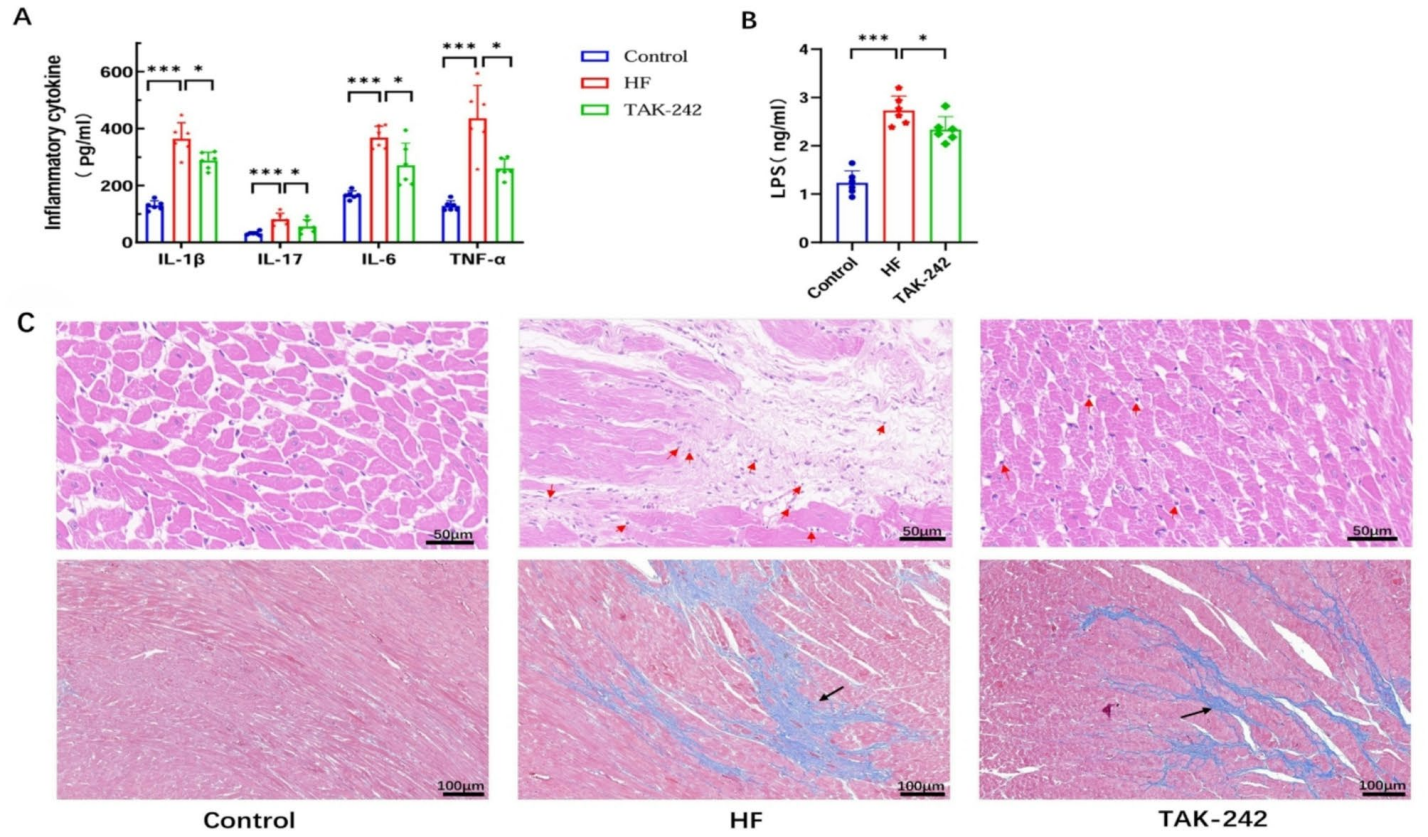
TAK-242 decreased serum inflammatory cytokines and LPS concentrations, and alleviated myocardial remodelling in heart failure. (**A**) Serum inflammatory cytokines concentrations, including IL-1β, IL-17, IL-6 and TNF-α were reduced by TAK-242 treatment. (**B**) LPS level was increased in HF group, while TAK-242 decreased LPS level. (**C**) Myocardial HE and Masson staining. In HE and Masson staining, heart failure rats exhibited disordered myocardial alignment, tissue swelling and hypertrophy, and lymphocyte infiltration, which were ameliorated by TAK-242 treatment. Lymphocytic infiltrations were indicated by red arrows. Myocardial fibroses were indicated by black arrows. *N* = 6 in each group, *P < 0.05, ***P < 0.001

Compared with control rats, HF rats presented significantly elevated serum levels of IL-1β, IL-17, IL-6, TNF-α, and LPS. After TAK-242 treatment, the LPS and inflammatory cytokine levels decreased, and cardiac function improved (Fig. 5A, B). HE and Masson staining (Fig. 5C) revealed orderly myocardial arrangement, clear nuclei, and distinct striations in control rats. In contrast, HF rats presented disordered myocardial alignment, tissue swelling and hypertrophy, and lymphocyte infiltration, which were ameliorated by TAK-242 treatment.

### TAK-242 inhibits TLR4/NF-κB signalling pathway expression

**Fig. 6:**
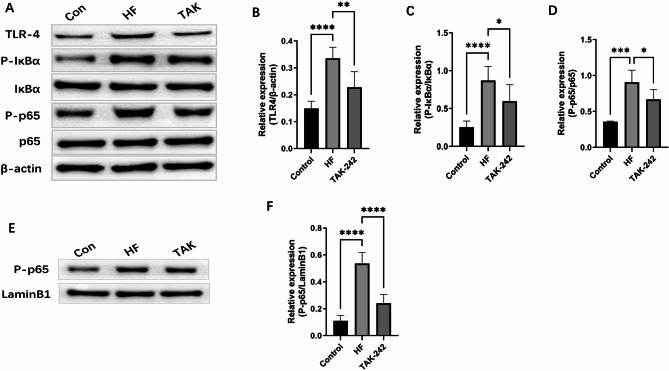
TAK-242 Inhibits TLR4/NF-κB Signalling Pathway Expression (**A**) Representative band images from immunoblot analysis of cytoplasmic protein expression in each group. The expression levels of TLR4 (**B**), P-IκBα/IκBα (**C**), and P-p65/p65 (**D**) in the myocardial cytoplasm were increased in the HF group, while TAK-242 treatment ameliorated these elevations. (**E**) Representative band images of nuclear protein P-p65 expression in each group. The expression of P-p65 in the myocardial nucleus was elevated in the HF group, whereas the TAK-242 group significantly reduced the expression of nuclear P-p65 (**F**). Data are presented as mean ± SD (*n* = 6 per group). *P < 0.05, **P < 0.01, ***P < 0.001. *P < 0.05, **P < 0.01, ***P < 0.001

Western blot analysis revealed significantly increased TLR4, P-IKBα/ IKBα and P-p65/p65 in HF rats compared with controls. TAK-242 treatment suppressed TLR4/NF-κB pathway activation, reduced TLR4 and P-p65/p65 expression. The expression of P-P65 in the nucleus was also significantly reduced after TAK-242 treatment (Fig. 6A-F).

## Discussion

HF is a complex clinical syndrome involving multiple pathophysiological processes. Although existing therapeutic strategies have improved the prognosis of patients to some extent, significant limitations remain. In recent years, the role of inflammation in HF has garnered increasing attention. Studies have indicated that dysregulation of the innate and adaptive immune systems plays a pivotal role in the initiation and progression of HF, leading to adverse cardiac remodelling, fibrosis, and dysfunction of cardiac and peripheral organs [27]. Through integrated multiomic analysis, this study systematically elucidated the molecular mechanism by which gut microbiota dysbiosis exacerbates HF by the LPS-TLR4/NF-κB signalling axis.

### Associations between gut microbiota dysbiosis and HF

The ISO hydrochloride-induced CHF rat model is a classical animal model for studying HF [28]. This study revealed significant gut microbiota restructuring in rats with ISO-induced HF. Specifically, the abundance of *Bacteroidota* (+ 38.7%) and *Spirochaetota* (+ 25.4%) increased, whereas that of *Actinobacteriot*a (− 42.1%) and *Proteobacteria* (− 31.6%) decreased (Fig. 3D-E). Concurrently, the Shannon diversity index decreased by 1.5-fold (*p* < 0.01). This microbiota imbalance resulted in a marked increase in the serum LPS level to 2.33 ± 0.53 ng/mL (vs. 0.98 ± 0.29 ng/mL in controls, *p* < 0.001), thereby activating the TLR4/NF-κB signalling pathway (TLR4 protein expression increased by 3.2-fold and p65 phosphorylation by 4.1-fold, *p* < 0.001) and driving excessive release of the proinflammatory cytokines IL-1β, IL-6, IL-17, and TNF-α (Fig. 5A-B).

Histopathological analysis further confirmed that the myocardial fibrosis area in the model group increased to 28.5 ± 3.7% (vs. 8.2 ± 1.5% in the control group, *p* < 0.001), and the LVEF significantly decreased to 53.6 ± 2.5% (vs. 81.3 ± 5.5% in the control group, *p* < 0.001), validating the pathological gut‒heart axis association (Fig. 5A and C).

### Regulatory role of the TLR4/NF-κB signalling pathway

Mechanistic investigations demonstrated that intervention with the TLR4 inhibitor TAK-242 not only suppressed p65 phosphorylation (− 73.2%, *p* < 0.001) and IκBα expression (2.1-fold, *p* < 0.01) but also reversed microbiota dysbiosis (*Bacteroidota* abundance was reduced to baseline ± 5.2%, *p* < 0.05), suggesting bidirectional regulation between TLR4 signalling and microbiota homeostasis (Fig. 3H-I). This finding transcends the limitations of traditional exogenous LPS stimulation models, revealing a novel mechanism by which endogenous microbiota metabolic disorders amplify cardiac inflammation by the LPS-TLR4/NF-κB cascade. Notably, TAK-242 treatment significantly improved cardiac function (LVEF increased to 61.9 ± 3.1%, *p* < 0.001) and reduced the fibrosis area to 12.1 ± 2.4% (*p* < 0.001). Its dual effects (inhibiting inflammation and modulating the microbiota) provide a theoretical basis for targeting the gut microenvironment in treating HF.

### Inflammatory mechanisms in the pathology of HF

Recent studies have increasingly indicated that chronic immune inflammation induces cardiomyocyte apoptosis and myocardial fibrosis, leading to impaired myocardial contraction and diastolic dysfunction, thereby triggering a cascade of HF symptoms [29, 30]. TLR4, a transmembrane protein in the TLR family, is highly expressed during cardiac injury and serves as a critical mediator of cardiac inflammation in cardiovascular disease progression [31]. Early studies revealed that TLR4 expression increases in cardiomyocytes during myocardial infarction-induced HF, promoting inflammatory responses and exacerbating HF [32]. In mice with LPS-induced sepsis, myocardial TLR4 and JNK protein expression, plasma TNF-α and cTnI levels, and cardiac dysfunction are elevated. Inhibiting TLR4 activation reduces JNK protein expression in cardiomyocytes and serum TNF-α levels, alleviating myocardial injury and improving cardiac function during sepsis [25]. Furthermore, TLR4/NF-κB pathway inhibition also ameliorates sepsis-induced cardiac dysfunction in rats [33].

### Interplay between the gut microbiota and HF

Significant differences exist in the gut microbiota composition between HF patients and healthy controls. Studies have reported greater colonization of pathogenic bacteria in the intestines of CHF patients, with 78.3% exhibiting increased intestinal permeability. Additionally, patients with moderate-to-severe congestive HF have greater intestinal permeability than those with mild congestion [34]. Heart failure induces intestinal barrier dysfunction, promoting LPS translocation into the bloodstream and increasing TLR4 expression [35]. In TLR4-knockout mice, high-fat diet-induced metabolic abnormalities—including impaired myocardial contraction, intracellular Ca2 + deficits, reactive oxygen species (ROS) accumulation, mitochondrial damage, inflammation, and autophagy—are mitigated, indicating that TLR4 knockout confers partial protection against high-fat diet-induced cardiac remodelling and systolic/diastolic dysfunction [36].

### Gut barrier function and microbial metabolism

Human intestinal epithelial cells are constantly exposed to vast bacterial populations and high concentrations of microbial metabolites. The intestinal mucosal barrier constitutes the first line of defence for the innate immune system against microbial invasion. Heart failure causes intestinal congestion, leading to microbiota dysmetabolism and bacterial translocation, resulting in a “leaky gut” phenomenon [37, 38]. Most relevant studies have employed exogenous high-dose LPS injections in rats to induce myocardial injury [39, 40]. The present study revealed that HF rats exhibited gut microbial dysbiosis at the phylum level, characterized by elevated abundance of *Bacteroidota* and *Spirochaetota*, which directly correlated with increased levels of proinflammatory mediators, including LPS, interleukin-1β (IL-1β), IL-6, IL-17, and TNF-α. TAK-242 intervention significantly reduced the relative abundance of *Bacteroidota* and *Spirochaetota*, concomitant with marked decreases in circulating LPS, IL-1β, IL-6, IL-17, and TNF-α levels, ultimately ameliorating cardiac dysfunction. Moreover, TAK-242 treatment induced genus-level microbial restructuring in HF rats, as evidenced by decreases in the abundance of *Muribaculaceae*, *Peptostreptococcaceae*, *Prevotellaceae*, and *Ruminococcaceae*, thereby rectifying HF-associated dysbiosis. Histopathological analysis revealed disordered myocardial arrangement, tissue thickening, swelling, and pronounced lymphocyte infiltration. However, treatment with the TLR4 antagonist TAK-242 markedly reduced LPS levels, decreased inflammatory cytokines, improved cardiac function, and alleviated myocardial disarray, tissue swelling, and lymphocyte infiltration. These findings suggest that alterations in the composition of the gut microbiota and changes in the concentration of the metabolite LPS in rats with HF cause changes in the levels of inflammatory factors in the blood, which contribute to altered cardiac function in these rats. However, a healthy intestinal flora is in dynamic balance, and *Bacteroidota* is a double-edged sword in the human intestinal system; Enterobacteriaceae can influence host growth by facilitating food digestion and nutrient acquisition [41]. *Bacteroidota* are the most abundant gram-negative bacilli in the human gut, and although they play an irreplaceable role in the intestinal flora between hosts, some anaplastic bacilli may be opportunistic pathogens [42, 43]. *Muribaculaceae* and *Prevotellaceae* have been identified as direct contributors to increased LPS levels [44]. Although *Peptostreptococcaceae* lacks intrinsic LPS-producing capacity, its overproliferation may indirectly promote LPS accumulation via disruption of intestinal barrier integrity or facilitation of proteobacterial proliferation [45, 46].

Notably, LPS, a major component of the outer wall of gram-negative bacterial cell walls, is a substance composed of lipids and polysaccharides and has been shown to be associated with systemic inflammation and chronic disease [47].

### Therapeutic potential of TAK-242

TAK-242, a small-molecule TLR4 inhibitor, is widely used to suppress TLR4/NF-κB pathway activation in studies of inflammation, cancer, and autoimmune diseases [48–50]. This study demonstrated that TAK-242 administration reduced serum LPS and inflammatory cytokine concentrations, reversed microbiota dysbiosis in HF rats, downregulated TLR4, P-IKBα and P-p65 protein expression in the TLR4/NF-κB pathway. Previous studies have indicated that NF-κB proteins in most resting cells remain inactive in the cytoplasm by binding to inhibitory IκB proteins. Upon pathway activation, IκB is degraded, releasing NF-κB into the nucleus to mediate transcriptional activation [51]. This study revealed that gut microbiota dysbiosis increases LPS production, enhances TLR4 binding, activates the TLR4/NF-κB pathway, and elevates IL-1β, IL-17, IL-6, and TNF-α levels, thereby inducing and aggravating HF in rats. TAK-242 improves gut microbiota dysbiosis and enhances cardiac function by blocking the TLR4/NF-κB pathway.

### Study limitations and future directions

Despite these significant findings, this study has several limitations: (1) the animal model does not fully replicate the progression of chronic HF in humans; (2) specific metabolic pathways underlying microbiota‒host interactions require further elucidation via metabolomics; and (3) whether TAK-242-mediated microbiota reprogramming operates independently of TLR4 inhibition remains to be validated. Future studies will integrate faecal microbiota transplantation and single-cell transcriptomics to delineate the molecular mechanisms by which specific bacterial genera (e.g., *Bacteroidota*) regulate cardiomyocyte pyroptosis via metabolites.

## Conclusion

This study demonstrates that gut microbiota dysbiosis during HF progression exacerbates cardiac dysfunction by activating the TLR4/NF-κB signalling pathway via endogenous LPS, thereby driving myocardial inflammation and fibrosis. The TLR4 inhibitor TAK-242 significantly improved cardiac function (LVEF increased by 15.5%, *p* < 0.001) and reduced the area of fibrosis (− 58.3%, *p* < 0.001) through a dual mechanism—suppressing p65 phosphorylation (− 73.2%) and restoring microbiota homeostasis (Shannon index increased by 1.8-fold). These findings not only enrich the theoretical framework of the “gut‒heart axis” but also provide a novel rationale for targeting the gut microenvironment in treating HF.

## Electronic supplementary material

Below is the link to the electronic supplementary material.

Supplementary Material 1

Supplementary Material 2

Supplementary Material 3

Supplementary Material 4

Supplementary Material 5

Supplementary Material 6

Supplementary Material 7

Supplementary Material 8

Supplementary Material 9

Supplementary Material 10

Supplementary Material 11

Supplementary Material 12

Supplementary Material 13

Supplementary Material 14

Supplementary Material 15

Supplementary Material 16

Supplementary Material 17

Supplementary Material 18

Supplementary Material 19

Supplementary Material 20

Supplementary Material 21

Supplementary Material 22

Supplementary Material 23

Supplementary Material 24

## Data Availability

The data underlying this article are available in the article and in its online supplementary material (10.6084/m9.figshare.28580111).
