# Correction: Gut microbiota dysbiosis exacerbates heart failure by the LPS-TLR4/NF-κB signalling axis: mechanistic insights and therapeutic potential of TLR4 inhibition

**DOI:** 10.1186/s12967-025-06943-z

**Published:** 2025-08-22

**Authors:** Chunlei Zhang, Xiaodong Teng, Qiuhang Cao, Yanyan Deng, Mo Yang, Lei Wang, Daorong Rui, Xiu Ling, Cao Wei, Yue Chen, Dasheng Lu, Hongxiang Zhang

**Affiliations:** 1 The Second Affiliated Hospital of Wannan Medical College Department of Cardiology, Wuhu, Anhui China; 2 Department of Respiratory Disease, Shangqiu Municipal Hospital, Shangqiu, Henan China; 3 Vascular Diseases Research Center of Wannan Medical College, Wuhu, Anhui China; 4 Wuhu Vascular Disease Technology Research and Development Center, Wuhu, Anhui China

**Correction to: Journal of Translational Medicine (2025) 23:762** 10.1186/s12967-025-06821-8.

In this article, corresponding authorship was not captured for Dasheng Lu due to a typesetting mistake.

The author group has been updated above and the original article has been corrected. The publisher apologises to the authors and readers for the inconvenience caused by this error.

